# Weight management in Canada: an environmental scan of health services for adults with obesity

**DOI:** 10.1186/1472-6963-14-69

**Published:** 2014-02-12

**Authors:** Marie-Michèle Rosa Fortin, Christine Brown, Geoff DC Ball, Jean-Pierre Chanoine, Marie-France Langlois

**Affiliations:** 1 Centre de recherche clinique Étienne-Le Bel, Centre Hospitalier Universitaire de Sherbrooke, Québec, Canada; 2 Department of Pediatrics, Faculty of Medicine and Dentistry, University of Alberta, Alberta, Canada; 3 Endocrinology and Diabetes Unit, British Columbia Children’s Hospital and University of British Columbia, British Columbia, Canada; 4 Department of Medicine, Division of Endocrinology, Faculté de médecine et des sciences de la santé, Université de Sherbrooke, Québec, Canada

**Keywords:** Obesity, Weight management, Bariatric surgery, Multidisciplinary team, Primary care

## Abstract

**Background:**

Obesity in Canada is a growing concern, but little is known about the available services for managing obesity in adults. Our objectives were to (a) survey and describe programs dedicated to weight management and (b) evaluate program adherence to established recommendations for care.

**Methods:**

We conducted an online environmental scan in 2011 to identify adult weight management services throughout Canada. We examined the degree to which programs adhered to the 2006 Canadian Clinical Practice Guidelines on the Management and Prevention of Obesity in Adults and Children (CCPGO) and the analysis criteria developed by the Association pour la Santé Publique du Québec (ASPQ).

**Results:**

A total of 83 non-surgical (34 community-based, 42 primary care-based, 7 hospital-based) and 33 surgical programs were identified. All programs encouraged patient self-management. However, few non-surgical programs adhered to the CCPGO recommendations for assessment and intervention, and there was a general lack of screening for eating disorders, depression and other psychiatric diseases across all programs. Concordance with the ASPQ criteria was best among primary care-based programs, but less common in other settings with deficits most frequently revealed in multidisciplinary health assessment/management and physical activity counselling.

**Conclusions:**

With more than 60% of Canadians overweight or obese, our findings highlight that availability of weight management services is far outstripped by need. Our observation that evidence-based recommendations are applied inconsistently across the country validates the need for knowledge translation of effective health services for managing obesity in adults.

## Background

The high prevalence of obesity in adults [1] and its serious health consequences [2-4] pose a major challenge for the Canadian health care system. The direct costs of overweight and obesity in Canada equaled six billion Canadian dollars in 2006, representing 4.1% of total health care expenditures [5]. In response, a national strategy on healthy living and prevention of chronic diseases was developed [6]. In parallel, many provinces have devoted resources to addressing obesity, mainly by increasing the availability of bariatric surgery [7-9]. To help guide obesity care, the Canadian Clinical Practice Guidelines on the Management and Prevention of Obesity (CCPGO) emphasize the fundamental role of multidisciplinary health care teams to effectively manage obesity [10].

Despite increased awareness of obesity, little is known about weight management services available to Canadian adults. Almost three-quarters of overweight and obese Canadian adults have tried to lose weight, but very few have reported receiving advice about weight management from health care providers [11]. This suggests that the health care system is not providing weight management services, which results in m any Canadians accessing the unregulated commercial weight-loss industry. The availability of commercial services varies substantially in quality, safety, and effectiveness, leading the Association pour la santé publique du Québec (ASPQ) to publish a reference guide outlining the fundamental principles that individuals can use to inform decision-making regarding weight management services [12]. Independently, a recent workshop of stakeholders from across Canada identified the completion of an inventory of health services for managing obesity as an important step to identify research priorities, policy directives, and resource allocation within the health care system [13]. With reference to this foundational work, the aims of this study were to (a) complete the first pan-Canadian environmental scan to identify programs for managing obesity in adults and (b) evaluate the degree to which programs adhere to recommendations from the CCPGO and ASPQ.

## Methods

### Study design

An online survey was created in collaboration with and hosted by the Canadian Obesity Network-Réseaucanadien en obésité (CON-RCO, ( http://con-initiatives.com/adultscan), which was based on previous research to quantify the number of pediatric weight management programs in Canada [14]. Our team used several strategies to ensure the survey was widely promoted at national level. For instance, all CON-RCO members (n >6500 as of 2011) received two email invitations in February and July 2011. Dr. Arya Sharma (CON-RCO Scientific Director) posted survey details on his blog in March 2011. The survey was advertised extensively during the 2nd National Obesity Summit, which was attended by over 800 obesity clinicians and researchers in Montreal, Quebec (May 2011). Invitations were distributed through 15 Canadian health care professional organizations (see acknowledgements) in early 2011. Finally, all individuals who completed the survey were encouraged to forward it to their colleagues to encourage recruitment through ‘word-of-mouth’.

All the program organizations were contacted by telephone or email between August and December 2011 to validate the information. For the sake of inclusivity, the definition of ‘weight management program’ was left to the discretion of program representatives. To calculate the number of programs per million of overweight or obese individuals, we divided the number of programs in each province by the number of persons who reported a body mass index (BMI) that classified them as overweight or obese in the CANSIM database (Statistics Canada, CANSIM, table 051-0001. Last modified: 2011-09-28).

### Program classification and rating

Non-surgical, publically funded programs as well as private, for-profit programs were categorized according to their setting. Three types of programs were classified: 1) a service offered in the community, e.g., group-based education and exercise sessions, was categorized as a ‘community-based program’, 2) a program that included individual assessment and counselling by a health care professional, e.g., physician and dietician, in the primary care environment was considered a ‘primary health care program’, and 3) a program based in a hospital was considered a ‘hospital-based program’. Any program that (i) had either a psychiatrist, a psychologist or a social worker on the health care team and (ii) reported offering psychological/emotional support services, e.g., stress management and emotional eating, or behavioral therapy was categorized as offering ‘psychological/behavioral counselling’. Commercial programs were invited to participate in the survey. They were classified in community-based or primary care programs depending on the presence of a physician in the team.

Programs were scored for their concordance with the CCPGO assessment recommendations according to the seven indicators presented in Table 1[10]. We also assessed the programs according to four of the seven ASPQ reference guide’s criteria that we were able to evaluate within the survey (Table 1) [12]. For example, a program promoting a credible rate of weight loss, e.g., less than 1 kilogram per week, or a credible weight management objective, e.g., 5 to 10% weight loss over six months, was considered adherent to the first criterion. The second criterion included two elements (the approach and the supervision). The ‘approach’ criterion was met for lifestyle modification or if food supplements or meal replacements were prescribed by a health professional. The ‘supervision’ criterion was met if the program was delivered by at least two health care professionals from different disciplines, e.g., physician and dietitian. A program offering nutritional counselling without the use of a long-term, very low energy diet, e.g., less than 1000 calories per day, adhered to the third criterion. Finally, a program offering physical activity counselling adhered to the fourth criterion.

**Table 1:** Criteria used to analyze the programs

**CCPGO assessment recommendations**	**ASPQ reference guide**’**s criteria**
Measuring body mass index (BMI)	Rate of weight loss
Measuring waist circumference	Approach required for a program or method (including supervision)
Assessing readiness to change	Dietary intervention
Completing medical history and physical examination	Physical activity counselling
Measuring fasting plasma glucose and determine lipid profile	Effectiveness of the approach ^#^
Performing additional metabolic investigations (liver enzymes tests, urine analysis, sleep studies)	Safety of the approach ^#^
Screening for eating disorders, depression and other psychiatric disorders	Promotion and advertising surrounding the approach ^#^
	Cost of the approach ^#^

Note:

# Data regarding these criteria were not reported since we lacked the data from many programs.

### Statistical analysis

Statistical analyses were performed with SPSS version 18.0 (SPSS Inc, Chicago, IL) and StatXact 3.1 (CytelInc, Cambridge, MA). Because all continuous variables were non-normally distributed, results are reported as median ± interquartile ranges (IQR, 25^th^–75^th^ percentiles). Categorical variables are reported as proportions. Chi-square tests or Fisher’s exact tests were used to examine differences between types of programs. A Bonferroni correction was used for multiple testing. We considered differences to be significant if the p-value was <0.05. The reported sample sizes (n) correspond to the number of programs for which information was available.

## Results

**Figure 1:**
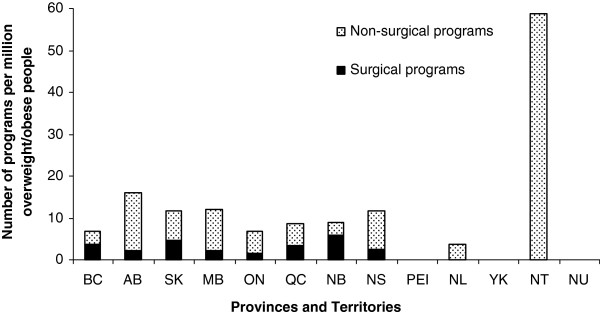
Number of programs per million of overweight or obese population in Canada in 2011*. (* Source: Statistics Canada, CANSIM, table 105-0501 and Catalogue no. 82-221-X.Last modified: 2012-06-19).

The online survey received 117 program entries from February to December 2011. In addition, our team manually entered data from nine surgical programs that did not complete the online survey, but were referred to us by the Canadian Association of General Surgeons Bariatric Surgery Working Group. Out of a total of 126 programs, ten were excluded (two were non-Canadian, three had duplicate entries, two non-surgical programs registered without completing the survey and could not be contacted for data verification, one was dedicated to animals, one included individuals exclusively with diabetes, and one program was discontinued before we were able to validate their information). The geographical distribution of the 116 remaining programs is shown in Table 2. The number of programs per million overweight or obese individuals in each province is shown in Figure 1, while the number of programs in relation to the total population of each province is shown in Additional file 1: Figure S1.

**Table 2:** Distribution of adult weight management services in Canada

**Provinces and territories**	**Programs exclusively surgical**	**Surgical programs including a medical component**	**Surgical programs-regional assessment treatment centers**	**Total surgical programs**	**Non-surgical community-based programs**	**Non-surgical primary health care programs**	**Non-surgical hospital-based programs**	**Total non-surgical programs**	**Total programs**
British Columbia	4	2	0	6	1	2	2	5	11
Alberta	2	1	0	3	10	10	0	20	23
Saskatchewan	1	1	0	2	1	2	0	3	5
Manitoba	1	0	0	1	4	1	0	5	6
Ontario	2	4	2	8	7	16	4	27	35
Quebec	4	6	0	10	9	7	0	16	26
New Brunswick	0	2	0	2	0	1	0	1	3
Nova Scotia	0	1	0	1	1	2	1	4	5
Newfoundland and Labrador	0	0	0	0	0	1	0	1	1
Northwest Territories	0	0	0	0	1	0	0	1	1
Total	14	17	2	33	34	42	7	83	116

### Program characteristics

Most programs were delivered at a single site (min = 1 site, max = 1502 sites; IQR 1–1 site, n = 112). The median time since the establishment of the program was five years (IQR 2–9.25 years, n = 107). Other program characteristics, e.g., source of funding and participation in research, are described in Additional file 2: Table S1.

### Program inclusion criteria

Programs had a variety of referral processes, including physician only (16%, n = 107), physician and/or other health care professional (16%, n = 107), and self-referrals (68%, n = 107). Only 33% (n = 24) of surgical programs accepted self-referrals; however, self-referral was very common among community-based programs (91%, n = 34). The median minimum patient age for referral was 18 years old (IQR 16–18 years old, n = 109) whereas the median patient maximal age for referral was 70 years old (IQR 65–80years old, n = 82), although approximately one-quarter (24%, n = 108) did not apply an upper age limit. Most programs used BMI as the primary inclusion criterion (Table 3) and applied exclusion criteria [surgical programs (100%, n = 27), community-based programs (71%, n = 34), primary health care programs (59%, n = 41) and hospital-based programs (86%, n = 7)], the most common being the presence of an active uncontrolled psychiatric disorder ,e.g., substance abuse, mental disease and eating disorder.

**Table 3:** Use of Body Mass Index (BMI) as an inclusion criterion by programs

**BMI (kg/m**^**2**^**) values accepted**	Surgical programs *, †, ‡	Non-surgical community-based programs *, §	**Non-surgical primary health care programs †, §**	**Non-surgical hospital-based programs ‡**
Not a criteria	0/26	20/34	12/42	1/7
	(0%)	(59%)	(29%)	(14%)
< 25	0/26	2/34	0/42	0/7
	(0%)	(6%)	(0%)	(0%)
25-29.9	0/26	9/34	24/42	4/7
	(0%)	(26%)	(57%)	(57%)
30-34.9	0/26	1/34	3/42	1/7
	(0%)	(3%)	(7%)	(14%)
≥35	2/26	1/34	1/42	0/7
	(8%)	(3%)	(2%)	(0%)
>30 or >27 with comorbidities	0/26	0/34	2/42	0/7
	(0%)	(0%)	(5%)	(0%)
>35 or >30 with comorbidities	3/26	0/34	0/42	0/7
	(12%)	(0%)	(0%)	(0%)
>40 or >35 with comorbidities	21/26	0/34	0/42	1/7
	(81%)	(0%)	(0%)	(14%)
Other (require Dr approval)	0/26	1/34	0/42	0/7
	(0%)	(3%)	(0%)	(0%)

Note: Data are expressed as number (%).

* Significant difference between surgical programs and community-based programs.

† Significant difference between surgical programs and primary health care programs.

‡ Significant difference between surgical programs and hospital-based programs.

§ Significant difference between community-based programs and primary health care programs.

### Patient assessment

Table 4 presents the assessment of patients according to the CCPGO recommendations. In general, community-based programs included the evaluation of fewer items than other categories of programs. Waist circumference (WC) was not measured systematically across all programs and screening for eating disorders, depression, and/or other psychiatric diseases was infrequently completed. Surgical programs tended to satisfy more of the recommendations (median score: 5/7) than non-surgical programs (median score: 3/7).

**Table 4:** Concordance of initial assessment with CCPGO recommendations

**Recommendation**		**Surgical programs**	**Non-surgical community-based programs**	**Non-surgical primary health care programs**	**Non-surgical hospital-based programs**
1-Measuring body mass index (BMI)		25/25	17/33	40/42	5/7
		(100%) *	(52%) *, §	(95%) §	(71%)
2-Measuring waist circumference		13/23	12/33	34/42	3/7
		(57%)	(36%) §	(81%) §	(43%)
3-Assessing readiness to change and motivation		18/23	23/33	36/42	5/7
		(78%)	(70%)	(86%)	(71%)
4-Clinical evaluation					
Medical history		25/25	18/34	40/42	5/7
		(100%) *	(53%) *, §	(95%) §	(71%)
General physical examination		25/25	2/32	21/42	2/7
		(100%) *, †, ‡	(6%) *, §	(50%) †, §	(29%) ‡
Both		25/25	2/32	21/42	1/7
		(100%) *, †, ‡	(6%) *, §	(50%) †, §	(14%) ‡
5-Screening tests					
Plasma glucose level		21/23	2/33	24/39	5/7
		(91%) *	(6%) *, §, ¶	(62 %) §	(71%) ¶
Lipid profile		21/23	2/33	25/39	5/7
		(91%) *	(6%) *, §, ¶	(64%) §	(71%) ¶
Both		20/23	2/33	24/39	5/7
		(87%) *	(6%) *, §, ¶	(62%) §	(71%) ¶
6-Additional investigations					
Liver function		21/23	1/33	18/39	4/7
		(91%) *, †	(3%) *, §, ¶	(46%) †, §	(57%) ¶
Urine analysis		15/24	1/33	13/42	0/7
		(63%) *, ‡	(3%) *, §	(31%) §	(0%) ‡
Sleep quantity/quality		22/24	14/34	30/42	4/7
		(92%) *	(41%) *, §	(71%) §	(57%)
All three		13/23	0/33	10/39	0/7
		(57%) *	(0%) *, §	(26%) §	(0%)
7-Screening for					
Eating disorders		15/23	6/33	24/42	2/7
		(65%) *	(18%) *, §	(57%) §	(29%)
Depression or other psychiatric diseases		15/23	4/33	23/42	2/7
		(65%) *	(12%) *, §	(55%) §	(29%)
Both		13/23	3/33	20/42	1/7
		(57%)	(9%) §	(48%) §	(14%)
**Median score**	**Out of 7 criteria**	**5**	**2**	**4**	**3**
		*	*, §	§	

Note: Data are expressed as number (%).

* Significant difference between surgical programs and community-based programs.

† Significant difference between surgical programs and primary health care programs.

‡ Significant difference between surgical programs and hospital-based programs.

§ Significant difference between community-based programs and primary health care programs.

¶ Significant difference between community-based programs and hospital-based programs.

### Types of intervention

**Figure 2:**
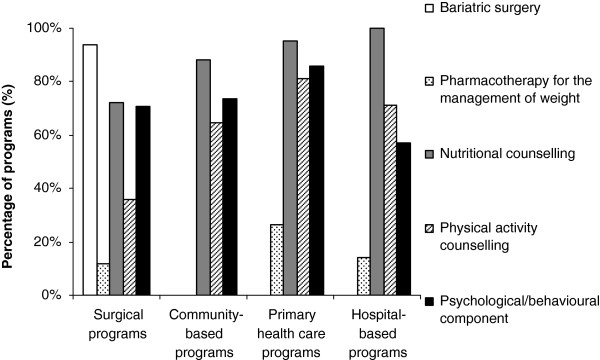
**Types of intervention offered by the different categories of programs.** Surgical programs n = 33 (for intervention components other than bariatric surgery n = 25 and for psychological/behavioral component n = 24), Community-based programs n = 34, Primary health care programs n = 42 and Hospital-based programs n = 7. Bariatric surgery = *, †, ‡. Pharmacotherapy for the management of weight = §. Nutritional counselling = none. Physical activity counselling = †. Psychological/behavioral component = none. *Significant difference between surgical programs and community-based programs. † Significant difference between surgical programs and primary health care programs. ‡ Significant difference between surgical programs and hospital-based programs. § Significant difference between community-based programs and primary health care programs.

Figure 2 describes the types of intervention offered. Bariatric surgery procedures performed by surgical programs (n = 29) included adjustable gastric banding (72%), sleeve gastrectomy (66%), Roux-en-Y gastric bypass (59%), biliopancreatic diversion with duodenal switch (10%), and intragastric balloon (7%). Most surgical programs (72%, n = 29) offered more than one type of procedure. Surgical programs offered less physical activity counselling than non-surgical programs (Table 5). Consistent with the CCPGO [10], support for self-management was provided by most programs [surgical programs (83%, n = 24), community-based programs (88%, n = 34), primary health care programs (98%, n = 42), and hospital-based programs (100%, n = 7)].

**Table 5:** Analysis of the programs according to selected ASPQ criteria

	**Surgical programs**	**Non-surgical community-based programs**	**Non-surgical primary health care programs**	**Non-surgical hospital-based programs**
Realistic rate of weight loss	Non applicable	30/31	35/36	3/3
		(97%)	(97%)	(100%)
Multidisciplinary assessment by health care professionals (≥ 2 types)	23/24	16/34	30/42	3/7
	(96%) *, ‡	(47%) *	(71%)	(43%) ‡
Nutrition counselling (without long-term use of very-low calorie diets)	18/25	29/34	34/42	5/7
	(72%)	(85%)	(81%)	(71%)
Physical activity counselling	9/25	22/34	34/42	5/7
	(36%) *, †	(65%) *	(81%) †	(71%)
**All**	**9/24**	**9/31**	**20/39**	**0/6**
	**(38%)**	**(29%)**	**(51%)**	**(0%)**

Note: Data are expressed as number (%).

*Significant difference between surgical programs and community-based programs.

† Significant difference between surgical programs and primary health care programs.

‡ Significant difference between surgical programs and hospital-based programs.

### Analysis according to selected ASPQ criteria

**Figure 3:**
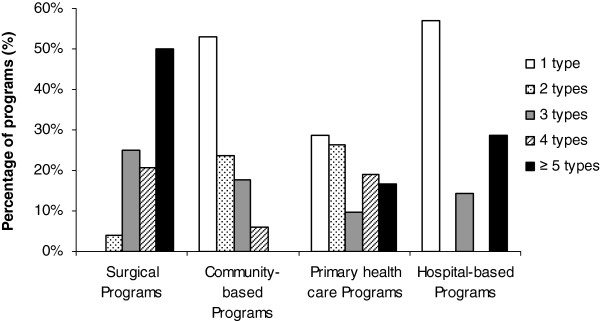
**Different types of professionals involved in weight management programs.** Surgical programs n = 24, Community-based programs n = 34, Primary health care programs n = 42 and Hospital-based programs n = 7. *, †, ‡. *Significant difference between surgical programs and community-based programs. † Significant difference between surgical programs and primary health care programs. ‡ Significant difference between surgical programs and hospital-based programs.

Few programs adhered to the four ASPQ criteria that we evaluated, but primary care programs rated the highest degree of adherence (Table 5). The main deficiencies included a lack of a multidisciplinary health care team and absence of physical activity counselling. Figure 3 shows the degree of program multidisciplinarity and Table 6 describes the types of health care professionals included on the teams. Surgical programs had a greater variety of team members and total number of health care professionals *versus* non-surgical programs. Community-based programs tended to include services that were provided by health care professionals working individually.

**Table 6:** Types of health care professionals involved in the programs

	**Surgical programs**	**Non-surgical community-based programs**	**Non-surgical primary health care programs**	**Non-surgical hospital-based programs**
Physician	23/24	2/34	21/42	2/7
	(96%) *, †, ‡	(6%) *, §	(50%) †, §	(29%) ‡
- General practitioner	4/24	2/34	16/42	0/7
	(17%)	(6%) §	(38%) §	(0%)
- Specialist	4/24	0/34	8/42	1/7
	(17%)	(0%)	(19%)	(14%)
- Internist	7/24	0/34	3/42	1/7
	(29%) *	(0%) *	(7%)	(14%)
- Surgeon	22/24	0/34	0/42	0/7
	(92%) *, †, ‡	(0%) *	(0%) †	(0%) ‡
Nurse	20/24	6/34	18/42	2/7
	(83%) *, †	(18%) *	(43%) †	(29%)
Mental health professional	16/24	8/34	20/42	3/7
	(66%) *	(24%) *	(48%)	(43%)
- Psychiatrist	7/24	0/34	1/42	0/7
	(29%) *, †	(0%) *	(2%) †	(0%)
- Psychologist	9/24	4/34	16/42	1/7
	(38%)	(12%)	(38%)	(14%)
- Social worker	10/24	5/34	5/42	2/7
	(42%) †	(15%)	(12%) †	(29%)
Dietitian	23/24	21/34	33/42	7/7
	(96%) *	(62%) *	(79%)	(100%)
Physical activity specialist	7/24	11/34	22/42	3/7
	(29%)	(32%)	(52%)	(43%)
CCPGO recommendation for the health care team ^#^	7/24	2/34	13/42	2/7
	(29%)	(6%) §	(31%) §	(29%)

Note:

# The CCPGO recommend that the multidisciplinary team should include a coordinating health professional (who may be a primary care physician, medical specialist or registered nurse), a dietitian, a physical activity specialist and a clinical psychologist. [10] We considered all categories of mental health professional suitable for the role of ‘clinical psychologist’.

Data are expressed as number (%).

* Significant difference between surgical programs and community-based programs.

† Significant difference between surgical programs and primary health care programs.

‡ Significant difference between surgical programs and hospital-based programs.

§ Significant difference between community-based programs and primary health care programs.

## Discussion

Our nationwide survey is the first comprehensive assessment of health services for managing obesity in Canadian adults. Overall, our research revealed several important findings. First, weight management programs in Canada are scarce, with approximately nine programs per million overweight or obese people. Second, the evaluation of the programs’ initial assessment and intervention according to *CCPGO* recommendations showed that (a) only primary health care programs systematically measure WC, (b) community-based programs tend to not complete physical examination and assessment of comorbidities compared with other types of programs, and (c) there is a lack of screening for mental health issues across all program categories. Third, most programs support their patients towards self-management. Finally, most programs did not fulfill the ASPQ criteria, with multidisciplinary assessment/management and physical activity counselling being the most deficient. However, primary care programs have the best concordance scores with the ASPQ criteria.

Few national reports have been published regarding health services available for managing obesity. For instance, one US-based report surveyed obesity treatment programs for both adults and children in public hospitals, which makes it difficult to directly compare with our study [15]. Nevertheless, the authors showed that the most frequent types of interventions for adults were nutrition-based (37.5%), clinic-based (35.0%), bariatric surgery (28%), primary care-based (20%), and research-based (17.5%). These findings are consistent with our observations. Compared with the recent survey of pediatric weight management programs in Canada [14], we found both a greater number and variety of services. This observation is most likely explained by the diversity of management settings available to the adult population compared with the pediatric population, which included mostly hospital-based services [14]. There were few hospital-based, non-surgical programs in our survey, suggesting that few hospitals are actively providing weight management outside of bariatric surgery. There is a need to develop this resource in the future to manage more complex cases, especially when surgery is not available or contraindicated.

Our results also show that although most programs (except community-based programs) measured patients’ BMI, only primary care programs systematically measured WC. For surgical programs, this might be explained by the fact that patients with severe obesity usually have a very high WC, and the measurement tends to be unreliable [16]. However, there is a need to focus on the WC measurement for adults, especially those with a BMI <35 kg/m^2^, which is consistent with the CCPGO recommendations [10]. In addition, there was a lack of screening of eating disorders, depression, and other psychiatric diseases in all categories of programs, including the surgical programs. This is surprising since patients with severe obesity have a high prevalence of depression [17,18], anxiety disorders [18] and eating disorders [19]. In addition, it is important to objectively assess the psychological health of individuals seeking bariatric surgery [20-22]. Nonetheless, a report by the National Confidential Enquiry into Patient Outcome and Death in the United Kingdom highlighted that only 29% of bariatric surgery patients had received any psychological input into their care, suggesting that even if bariatric surgery programs offer a psychological/behavioral component, few patients complete an assessment or receive care [23]. Our results are also in line with a recent Omnibus survey that indicated the current obesity guidelines are still not effectively implemented [11]. Although numerous interventions have been shown to be effective [24-26], guidelines are still applied inconsistently in routine care by health professionals because of barriers such as time constraints, lack of awareness or familiarity with the guidelines, disagreement with the guidelines’ conclusions or relevance, lack of resources and support, or lack of motivation to work with patients with obesity [27-29]. Finally, the majority of programs did not adhere to the criteria recommended by ASPQ [12]. However, primary care-based programs adhered most closely to these criteria, which is encouraging, since this is where most Canadians access the health care system. Furthermore, as others have shown [30], there was a lack of physical activity counselling in the surgical programs. This is surprising since it appears that exercise may help weight loss after the surgery [31-33] and that physical activity favors weight maintenance [34-36].

Some limitations must be taken into consideration when interpreting our data. First, despite using a variety of methods to ensure our survey was circulated broadly throughout Canada, some programs may not have been surveyed. Second, our results relied on self-reported data, although our efforts to validate the data using follow-up telephone interviews with program representatives minimized this bias. Third, programs that could not be reached by telephone for validation had some missing data. Nevertheless, the use of a standardized questionnaire allowed us to compare programs. It should also be noted that some criteria of concordance with CCPGO recommendations, e.g., assessing readiness to change and realistic weight loss goals, lack empirical validation. We chose to report ‘programs per million overweight or obese people’, but this metric does not capture the number of people served per million since a program may serve 13 or 40,000 patients a year. Finally, not all the ASPQ criteria were applied because of the lack of data, e.g. costs and advertising.

## Conclusions

Although high-quality weight management services are offered in academic, public, and private practices in Canada, the supply of services is vastly outstripped by potential demand across the country. Few non-surgical programs follow the CCPGO recommendations for assessment, especially for screening of psychiatric or eating disorders. In addition, the majority of programs do not adhere to the ASPQ criteria, with multidisciplinary assessment/management and physical activity counselling being the most deficient. This national perspective can help to inform clinicians and administrators within the Canadian health care system to understand existing strengths, as well as address areas of weakness that require additional planning and implementation to optimize obesity-related health services for adults with obesity.

## Competing interests

The authors declare that there have no competing financial interests. The project was supported by grants from the Canadian Institutes of Health Research (CIHR, Partnerships for Health Systems Improvement, PHE-103970) and the Ministère de la santé et des services sociaux du Québec. The Centre de rechercheclinique Étienne-Le Bel is an FRQ-S funded research center. At the time of this research, Geoff Ball was a Population Health Investigator with Alberta Innovates – Health Solutions and a New Investigator of CIHR. Marie-France Langlois is the recipient of National researcher award from the Fonds de la recherche du Québec - Santé (FRQ-S).

## Authors’ contributions

All of the authors contributed to the conception of the study. Marie-Michèle Rosa-Fortin performed all phone interviews, conducted the analysis and interpretation of data and drafted the manuscript. Marie-France Langlois and Christine Brown also participated in data interpretation. All of the authors contributed to critically revise the manuscript and approved the final version submitted for publication.

## Pre-publication history

The pre-publication history for this paper can be accessed here:

http://www.biomedcentral.com/1472-6963/14/69/prepub

## Supplementary Material

Additional file 1: Figure S1 Number of programs per million of population in Canada in 2011* (* Source: Statistics Canada, CANSIM, table 051-0001. Last modified: 2011-09-28.) Click here for file

Additional file 2: Table S1 Program characteristics. Click here for file
