# Supplementary material for: Weight management in Canada: an environmental scan of health services for adults with obesity
Source: BMC Health Serv Res. 2014 Feb 12;14:69. doi: 10.1186/1472-6963-14-69 (PMC3927222; doi:10.1186/1472-6963-14-69)
Supplement: Additional file 1: Figure S1 — Number of programs per million of population in Canada in 2011* (* Source: Statistics Canada, CANSIM, table 051-0001. Last modified: 2011-09-28.) [file 1472-6963-14-69-S1.doc]

Additional file 1: Figure S1
