# Supplementary material for: Weight management in Canada: an environmental scan of health services for adults with obesity
Source: BMC Health Serv Res. 2014 Feb 12;14:69. doi: 10.1186/1472-6963-14-69 (PMC3927222; doi:10.1186/1472-6963-14-69)
Supplement: Additional file 2: Table S1 — Program characteristics. [file 1472-6963-14-69-S2.doc]

**Additional file 2: Table S1.** Program characteristics

|  | **Surgical programs** | **Non-surgical community-based programs** | **Non-surgical primary health care programs** | **Non-surgical hospital-based programs** |
| --- | --- | --- | --- | --- |
| Affiliated | 20/25  (80%) * | 14/34  (41%) * | 21/42  (50)% | 7/7  (100%) |
| - With an academic institution and/or a hospital | 18/25  (72%) *, † | 3/34  (9%) *, ¶ | 9/42  (21%) † | 5/7  (71%) ¶ |
| Funding |  |  |  |  |
| - Public funding only | 12/27  (44%) | 10/34  (29%) | 7/41  (17%) | 4/7  (57%) |
| - Fee-for-service only | 4/27  (15%) † | 14/34  (41%) | 26/41  (63%) †, ** | 0/7  (0%) ** |
| - Mixed funding | 11/27  (41%) | 10/34  (29%) | 8/41  (20%) | 3/7  (43%) |
| Program evaluation | 10/23  (43%) | 21/34  (62%) | 20/42  (48%) | 2/7  (29%) |
| - For efficacy | 6/23  (26%) | 11/34  (32%) | 12/42  (29%) | 1/7  (14%) |
| Research involvement | 15/24  (63%) *, † | 6/34  (18%) * | 10/42  (24%) † | 3/7  (43%) |

Note: Data are expressed as number (%)

* Significant difference between surgical programs and community-based programs

† Significant difference between surgical programs and primary health care programs

‡ Significant difference between surgical programs and hospital-based programs

§ Significant difference between community-based programs and primary health care programs

¶ Significant difference between community-based programs and hospital-based programs

** Significant difference between primary health care programs and hospital-based programs
